# A Comparison of the Self-Perceived Organisational and Social Work Environment among Swedish Occupational Therapists in Different Job Sectors: An Observational Study

**DOI:** 10.3390/ijerph20043009

**Published:** 2023-02-09

**Authors:** Elisabeth Dahlbäck, Carita Håkansson

**Affiliations:** 1 Occupational and Environmental Medicine, Medicon Village, 223 81 Lund, Sweden; 2 Division of Occupational and Environmental Medicine, Lund University, Medicon Village, 223 81 Lund, Sweden

**Keywords:** job sector, mental health, occupational therapist, organisational and social work environment

## Abstract

Sick leave due to mental health problems is increasing, and there is evidence that it is associated with the individual’s self-perceived organisational and social work environment. The aim of this study was to compare occupational therapists’ self-perceived organisational and social work environments in different job sectors. The goal is to identify the sectors with the most unfavourable work environment and thus where the need to improve the work environment, to prevent mental health problems, is greatest. A web survey was emailed to working members of the Swedish Association of Occupational Therapists in February 2018 (*n* = 7600). The response rate was 48% (*n* = 3658). Studied job sectors were somatic specialist health care; elderly care; habilitation; psychiatric health care; primary health care; and university (*n* = 2648). This sample is representative of Swedish occupational therapists with respect to age, gender, and job sector. The web survey included questions on their sociodemographic characteristics and self-perceived organisational and social work environment regarding workload, control, community in the workplace, reward, justice, and values. Questions on the self-perceived organisational and social work environment were assessed by the QPS mismatch questionnaire. Differences in work environmental conditions between the job sectors were tested with ANOVA and post hoc multiple-group analysis. The results showed that occupational therapists working in psychiatric health care perceived the highest proportion of unfavourable working conditions. Occupational therapists who worked at universities perceived a higher workload than occupational therapists in most of the other studied job sectors. These job sectors need to be specifically addressed with adjustments to prevent mental health problems.

## 1. Introduction

Sick leave due to mental health problems is a growing challenge in Sweden [1] and the most common cause of sick leave in women of all ages and men under 60 years of age, as of December 2020 [2]. Mental health problems such as exhaustion lead to, among other symptoms, sleep disturbances and impaired self-perceived memory [3], which impact not only the individual but also the quality of their work [4]. Work dominates everyday life, and not least the organisational and social work environment affects employees’ mental health [5,6]. In order to be able to identify the measures that need to be taken to promote a good organisational and social work environment and thus prevent mental health problems, it is important to compare the work environment in different job sectors. Individuals working in the public sector are at high risk of sick leave due to mental health problems [1]. In this study, we therefore, focus on the self-perceived organisational and social work environment of occupational therapists in different job sectors.

An organisational work environment consists of conditions such as responsibilities, autonomy, the structural arrangement of work tasks, resources, and leadership. A social work environment, on the other hand, comprises circumstances such as interactions, collaborations, and social support from colleagues and managers [7]. 

This study is part of a research project that has thus far shown that occupational therapists perceive difficulties in doing a good job due to a high workload and increased stress. In the project, 3658 Swedish occupational therapists, responded to a web survey and almost 20 percent reported having symptoms related to different stages of exhaustion disorder. Moreover, nearly 60 percent reported having seriously considered seeking new employment during the last year, and about 35 percent reported having seriously intended to leave their profession during that same period [8]. Additionally, in the project, stress and high work pressure have been shown to be significant reasons for occupational therapists to consider leaving their profession [9]. Correspondingly, factors such as low workload, high control, high sense of community, and high justice in the organisational and social work environment have been shown to be associated with no or negligible stress symptoms [5]. The project has also revealed that the majority of occupational therapists with less than 10 years of experience report high control and high social support in their organisational and social work environment [10]. 

Occupational therapists’ organisational and social work environment has mainly been studied in countries other than Sweden. The results of these studies showed that increasing workload, working overtime, feeling pressure to discharge, balancing competing priorities and unrealistic demands, a mismatch between professional values and demands of employers, lack of respect from other members of the multidisciplinary team, lack of autonomy in the ability to customise their practice, and lack of health care resources affected the organisational and social work environment negatively [11]. Furthermore, invested effort and rewards received were significant aspects influencing the organisational and social work environment [12]. Contact with users and their families was highlighted as a significant factor influencing the organisational and social work environment [13]. The management structuring their work, and opportunities for occupational therapists to improve their skills, were significant aspects influencing the organisational and social work environment [14]. The size of the hospital has also been shown to be a factor that significantly influences the organisational and social work environment [15].

Occupational therapists work in many different job sectors such as somatic specialist health care, psychiatric health care, primary health care, habilitation, and elderly care, as well as at universities. The existing literature illustrates the situation regarding occupational therapists’ self-perceived organisational and social work environment in a general view. To the best of our knowledge, there are no previous studies on how the self-perceived organisational and social work environments vary in different job sectors among occupational therapists. 

The aim of this study was, accordingly, to compare the self-perceived organisational and social work environment regarding workload, control, community, reward, justice, and values in different job sectors. 

## 2. Materials and Methods

This study was part of a research project concerning occupational health among Swedish occupational therapists. It is based on a web survey analysed by means of statistical methods. Data was handled anonymously with a secure web application.

### 2.1. Participants

The criterion for the selection of participants was being a working member of the Swedish Association of Occupational Therapists as of February 2018. This organisation is both a trade union and a professional organisation with responsibility for promoting the occupational therapy profession. About 75 percent of Swedish occupational therapists are members of it. The exclusion criteria were being on sick leave, parental leave, unemployed, or retired. In February 2018, a web survey was forwarded to the therapists who met the inclusion criterion (*n* = 7600), and 3658 responded, giving a response rate of 48 percent. In this study, data from participants working in six different job sectors, was included (*n* = 2648).

### 2.2. Data Collection

The web survey included mainly validated questionnaires on the work environment, work–life balance, physical activity, and health, but also questions on sociodemographic characteristics. The survey comprise 117 questions. 

In the present study, questions on organisational and social work environment and sociodemographic characteristics including age, gender, occupational therapy education degree, work experience in years, degree of employment, and job sector were used. Questions relating to the participants’ self-perceived organisational and social work environment were assessed by the QPS mismatch questionnaire. The QPS mismatch questionnaire measures the self-perceived experience of risk factors in the organisational and social work environment which, according to the questionnaire, comprise (1) workload, (2) control, (3) community in the workplace, (4) reward, (5) justice, and (6) values. Each item is rated on a 5-point scale. For more detailed information on the specific organisational and social work environment factors, as defined by the questionnaire, see Table 1. QPS mismatch is a shortened version of the internationally and nationally validated QPS Nordic general questionnaire for psychological and social factors at work [16]. The development of the shorter QPS mismatch is intended to provide an approximate picture of the individuals’ mismatch between psychosocial working conditions and his or her ability. The reliability was shown to be good (Cronbach alpha 0.72–0.90 in all dimensions) [Österberg, 2016, personal communication]. Data were collected and handled with the secure web application Research Electronic Data capture (REDcap). A link to the web survey was sent to e-mail addresses collected from the member list of the Swedish Association of Occupational Therapists. The survey was accessible for three weeks and a reminder was sent on two occasions.

The questionnaire requested that the occupational therapists specify their current job sector, with 20 different job sectors to choose from. No differentiation was made between the public or private sectors in the questionnaire. The job sectors were grouped according to the field of activity they are part of.

- Somatic specialist health care (*n* = 572)
- Elderly care (*n* = 1070)
- Habilitation (*n* = 265)
- Psychiatric health care (*n* = 278)
- Primary health care (*n* = 387)
- University (*n* = 76)

### 2.3. Statistical Analysis

For each risk factor (workload, control, community in the workplace, reward, justice, and values) a mean score, in accordance with the User’s Guide for QPS Nordic [17], was calculated, and the differences between the job sectors were tested in the first step by variance analysis (ANOVA). In the next step of the analysis, which separated and compared each of the respective job sectors, a post hoc test (Bonferroni) was used. The choice of variance analysis and post hoc test was due to the exploratory nature of the research and reflected the research aim of identifying group differences in risk factors rather than identifying outcome variable subsets or underlying constructs associated with the outcomes. Data analysis was performed using SPSS Statistics 25.0.

## 3. Results

Most participants in the study were women and middle-aged. The majority worked more than 75 percent of full-time and had between 11 and 30 years of experience working as an occupational therapist. For more detailed information on sociodemographic factors, see Table 2. For information regarding mean values and standard deviations of the work characteristics in different job sectors, see Table 3.

### 3.1. Workload

The results indicated a significant difference in self-perceived workload among the job sectors F 13.4 (5, 2414) *p* < 0.001. The sectors with the lowest mean values, indicating more unfavourable self-perceived work environment conditions concerning self-perceived workload (distribution of work tasks over time, degree of distracting disturbances, degree of work obligations distressing personal life, and complaint handling), were adult habilitation and university. Pairwise comparisons of the means revealed significant differences between habilitation and somatic specialist health care (*p* < 0.001), habilitation and elderly care (*p* < 0.001), habilitation and psychiatric health care (*p* < 0.001), university and somatic specialist health care (<0.001), university and elderly care (*p* = 0.002), and university and psychiatric health care (*p* < 0.001).

Subsequently, in primary health care, the mean value self-perceived regarding workload was statistically significantly lower than in somatic specialist health care (*p* < 0.001) and psychiatric health care (*p* = 0.012). Standing out in a positive manner was somatic specialist health care. This sector had a statistically significantly higher mean value, indicating a more favourable self-perceived work environment concerning workload than all other studied sectors. In addition, psychiatric health care had a statistically significantly higher mean value when compared to habilitation (*p* < 0.001), primary health care (*p* = 0.012), and university (*p* < 0.001). In elderly care, the mean value presented a statistically significantly more favourable workload than habilitation (*p* < 0.001) and university (*p* = 0.002). On the contrary, it showed a statistically significantly more unfavourable self-perceived workload when compared to somatic specialist health care (*p* = 0.002).

### 3.2. Control

The results indicated a significant difference in self-perceived control among the job sectors F 4.15 (5, 2413) *p* = 0.001. Regarding self-perceived control (goals and areas of responsibility, the ability of the occupational therapists to impact their workload, the ability of the occupational therapists to impact their work rate, the ability of the occupational therapists to impact resource allocation, and the ability of the occupational therapists to impact their decisions), the lowest mean value indicating more unfavourable conditions was in elderly care. The results were only statistically significant when compared to somatic specialist health care (*p* < 0.001).

### 3.3. Reward

The results indicated a significant difference in self-perceived reward among the job sectors F 4.41 (5, 2394) *p* = 0.001. Concerning self-perceived reward (appreciation from managers, salary, and encouragement), the lowest mean value indicating more unfavourable conditions was in elderly care. The result was statistically significant only when compared to somatic specialist health care (*p* = 0.003).

### 3.4. Community in the Workplace

The results indicated a significant difference in the self-perceived community in the workplace among the job sectors F 5.0 (5, 2416) *p* < 0.001. Regarding self-perceived community in the workplace (support from colleagues and managers, occurrence of conflict at the workplace, and the environment concerning communication), psychiatric health care stood out in a negative manner with the lowest mean value, demonstrating more unfavourable conditions. This was statistically significant when compared to all other studied sectors (somatic specialist health care *p* = 0.023, elderly care *p* < 0.001, habilitation *p* = 0.002, primary health care *p* = 0.002) except university, where there was no statistically significant difference. 

### 3.5. Justice

The results indicated a significant difference in self-perceived justice among the job sectors F 5.8 (5, 2406) *p* < 0.001. Regarding self-perceived justice (impartiality from managers, willingness from managers to prioritise the handling of obstacles), the lowest mean value indicating more unfavourable conditions was in psychiatric health care. The result was statistically significant when compared to somatic specialist health care (*p* < 0.001), habilitation (*p* = 0.04), and primary health care (*p* = 0.002).

### 3.6. Values

The results indicated a significant difference in self-perceived values among the job sectors F 10.2 (5, 2414) *p* < 0.001. Regarding self-perceived values (conflicts between working tasks and personal values, conflicts between personal values and the values of the workplace, trust in the management’s ability to handle the future of the workplace, and willingness to recommend the workplace to a friend), the lowest mean value indicating more unfavourable conditions was in psychiatric health care, and the results were statistically significant when compared to all other studied sectors (somatic specialist health care (*p* < 0.001), elderly care *p* < 0.001), habilitation (*p* = 0.007), primary health care (*p* < 0.001), university (*p* < 0.001)). On the contrary, universities stood out in a positive manner regarding values. This sector has the highest mean value showing more favourable conditions concerning these factors than nearly all other sectors (somatic specialist health care (*p* = 0.002), elderly care *p* < 0.001), habilitation (*p* < 0.001), primary health care (*p* = 0.002)).

## 4. Discussion

The overall aim of this study was to compare the self-perceived organisational and social work environment in Swedish occupational therapists regarding workload, control, community in the workplace, reward, values, and justice in different job sectors. To the best of our knowledge, this is the first study that compares how occupational therapists rate their social and organisational work environment differently in multiple job sectors. The study showed statistically significant differences in all aspects of the self-reported organisational and social work environment between the studied job sectors. The study moreover identified specific job sectors that, compared to the other studied job sectors, stand out with more unfavourable self-reported working conditions.

Occupational therapists working within the field of psychiatric health care were marked as the group with the highest proportion of unfavourable self-reported working conditions. This group had the lowest mean in three of the six studied working aspects of organisational and social working conditions, indicating an unfavourable work environment regarding community in the workplace, justice, and values, and the results are statistically significant compared to the majority of the other studied groups. According to previous studies, unfavourable working conditions indicate possible risk factors for mental health problems [5,6]. International studies have, moreover, shown that well-being at work in occupational therapists working in the mental health sector is associated with turnover intention [12,13,18]. In addition, previous studies have shown that work-related well-being and satisfaction in occupational therapists working in mental health can be improved, if efforts are made to increase the meaningfulness of work activities by engaging the occupational therapists in duties they find valuable and by rewarding them through payment and appreciation [12,13,18]. This job sector thus needs to be addressed, with its working conditions examined further and interventions made to enhance more favourable working conditions and mental health.

Occupational therapists who work within the field of university graded their self-perceived workload more unfavourably than occupational therapists in most of the other studied job sectors, and the findings are statistically significant. As a previous study has shown that Swedish occupational therapists, in general, rate their workload as high [8]; this result presumably points to a high degree of workload within this job sector, and this result should, therefore, be taken seriously. The finding of a high workload among occupational therapists who work in the academic field is in line with previous findings on academic staff. High workload and a high degree of stress within university academics have been widely reported internationally, and a recent review article on the mental health of university academics has concluded that the university environment, with high expectations for academics in teaching, supervising, and generating research income, triggers high levels of stress and burnout and low levels of wellbeing [19]. In the current study, both contrarily and encouragingly, this group of occupational therapists rated their social and organisational work environment regarding values statistically significantly more favourable compared to all other studied groups. 

Although the results of this study showed statistically significant differences in the organisational and social work environment between the job sectors, we cannot assess whether the differences are significant in practice. However, the results of this study can be interpreted as if there is a need to promote a good organisational and social work environment and prevent mental health problems among Swedish occupational therapists. Different workplace strategies can be used in this work [20]. One strategy can be to design work that minimises harm, such as flexible working conditions [21]. Other strategies can be manager and leadership training [22] and mental health education [23], but also individual strategies, such as stress management programs [24] or coaching [25], can be recommended.

### Strengths and Limitations

This is the first study that has investigated and identified differences in the self-perceived organisational and social work environment among occupational therapists in different job sectors in Sweden. A limitation of the study is the design, which limits the possibility to draw conclusions on the causes of the differences based on the results. There is, therefore, a need for further research with a longitudinal design, exploring the factors predicting the self-perceived social and organisational work environment. The response alternatives of the QPS mismatch are a Likert scale, i.e., an ordinal scale, and the distance between responses is not measurable. Thus, the differences between the response alternatives, such as never, seldom, and sometimes, are not necessarily equal. That is, the statistically significant differences between the different job sectors should be interpreted with some caution. The QPS mismatch questionnaire is based on the well-validated QPS Nordic questionnaire, but a limitation is that the psychometric properties of the QPS mismatch have not been evaluated, which may decrease the internal validity of the present study. Another limitation is that the QPS mismatch has only been used in this research project and the results of this study cannot be discussed in relation to the results of other studies.

The response rate (48%) is similar to the response rate in other large-scale surveys targeting the general population [26], police employees [27], and principals [28], and enhances the credibility of the results. A strength is that this sample is representative of Swedish occupational therapists with respect to age, gender, and job sector (personal communication of Åsa Ehinger, the Swedish Occupational therapist association).

## 5. Conclusions

There are statistically significant differences in the self-perceived organisational and social work environments of the studied job sectors that employ occupational therapists. 

Some job sectors stand out as having more unfavourable working conditions. The sector consisting of psychiatric health care has the highest proportion of unfavourable self-perceived working conditions. 

By pinpointing job sectors where occupational therapists rate a higher degree of self-perceived unfavourable working conditions, this study identifies job sectors where the need for future adjustments and targeted intervention is greatest. Additionally, the study identifies areas where future qualitative research could, by exploring the experiences of the occupational therapists through interviews and observing their workplace, contribute to more knowledge about potentially harmful organisational and social working conditions. 

## Figures and Tables

**Table 1:** Risk factors in the organisational and social work environment as defined by the questionnaire.

Workload	Distribution of work tasks over time Degree of distracting disturbances Degree of work obligations distressing personal life Complaint handling
Control	Goals and areas of responsibility The ability of the occupational therapists to impact their workload The ability of occupational therapists to impact their work rate The ability of occupational therapists to impact resource allocation The ability of occupational therapists to impact their decisions
Community in the workplace	Support from colleagues and managers Occurrence of conflicts at the workplace Environment concerning communication
Reward	Appreciation from managers Salary Encouragement
Justice	Impartiality from managers Managers’ willingness to prioritise the handling of obstacles
Values	Working tasks in conflict with personal values Would recommend my workplace to a friend My personal values are comparable to the values of the workplace Trust in the management’s ability to handle the future of the workplace

**Table 2:** Sample demographics (*n* = 2648).

	*n*	(%)
Gender ^1^		
Female	2509	95.1
Male	124	4.7
Other	5	0.2
Age groups (years) ^2^		
≤29	315	12.0
30–39	613	23.4
40–49	704	26.9
50–59	695	26.6
≥60	288	11.0
Experience as an occupational therapist (years) ^3^		
≤10	864	33.1
11–19	705	27.0
20–29	614	23.5
30–39	373	14.3
≥40	58	2.2
Percentage of hours worked full-time ^4^		
≤75%	276	11.0
≥76%	2228	89.0

^1^ Missing *n* = 10, ^2^ Missing *n* = 33, ^3^ Missing *n* = 34, ^4^ Missing *n* = 144.

**Table 3:** Work characteristics of the different job sectors by mean (SD).

Work Characteristics	M (SD)
*Favourable workload*	
Somatic specialist health care	3.32 (0.68)
Elderly care	3.17 (0.68)
Habilitation	2.97 (0.76)
Psychiatric health care	3.27 (0.74)
Primary health care	3.08 (0.71)
University	2.84 (0.77)
*Control*	
Somatic specialist health care	3.50 (0.58)
Elderly care	3.37 (0.60)
Habilitation	3.39 (0.57)
Psychiatric health care	3.46 (0.60)
Primary health care	3.39 (0.58)
University	3.49 (0.58)
*Reward*	
Somatic specialist health care	2.67 (1.01)
Elderly care	2.46 (1.00)
Habilitation	2.49 (0.93)
Psychiatric health care	2.47 (1.04)
Primary health care	2.63 (1.02)
University	2.78 (1.06)
*Community in the workplace*	
Somatic specialist health care	3.82 (0.63)
Elderly care	3.86 (0.68)
Habilitation	3.88 (0.59)
Psychiatric health care	3.66 (0.67)
Primary health care	3.97 (0.64)
University	3.68 (0.72)
*Justice*	
Somatic specialist health care	3.34 (0.83)
Elderly care	3.20 (0.84)
Habilitation	3.27 (0.78)
Psychiatric health care	3.05 (0.86)
Primary health care	3.30 (0.80)
University	3.37 (0.80)
*Values*	
Somatic specialist health care	3.46 (0.74)
Elderly care	3.40 (0.76)
Habilitation	3.40 (0.73)
Psychiatric health care	3.17 (0.84)
Primary health care	3.45 (0.76)
University	3.84 (0.70)

## Data Availability

The data have been archived according to the Swedish Act concerning the Ethical Review of Research involving Humans and do not include data sharing. A minimal data set could be shared upon request, provided the data transfer is in agreement with EU legislation on the general data protection regulation and approved by the Swedish Ethical Review Authority.
